# Dual-Function Role of Phenolated Albumin in Hemin-Mediated Hydrogel Formation

**DOI:** 10.3390/gels11110912

**Published:** 2025-11-15

**Authors:** Shinji Sakai, Yuki Kitatani, Maasa Shiba, Thotage Asanka Vishwanath, Kelum Chamara Manoj Lakmal Elvitigala, Wildan Mubarok, Kousuke Moriyama

**Affiliations:** 1 Department of Materials Engineering Science, Graduate School of Engineering Science, The University of Osaka, Toyonaka 560-8531, Japanasanka.vish@cheng.es.osaka-u.ac.jp (T.A.V.); kelum@cheng.es.osaka-u.ac.jp (K.C.M.L.E.); wildanmubarok@cheng.es.osaka-u.ac.jp (W.M.); 2 Department of Chemical and Biological Engineering, National Institute of Technology, Sasebo College, 1-1 Okishin-cho, Sasebo 857-1193, Japan

**Keywords:** hemin, hydrogel, albumin, crosslinking, hydrogen peroxide

## Abstract

Enzymatically crosslinked hydrogels are important in biomedical applications. However, conventional horseradish peroxidase (HRP)-based systems are expensive, unstable, and potentially immunogenic. Herein, we introduce hemin/albumin complexes as cost-effective and biocompatible catalysts for phenol-mediated hydrogel formation. Phenolated bovine serum albumins (BSA-LPh, -MPh, and-HPh) with different degrees of substitution were synthesized and complexed with hemin. Spectroscopic analysis demonstrated that phenol modification altered the hemin microenvironment, resulting in distinct shifts in the Soret band. Functional assays revealed that albumin complexation enhanced catalytic activity compared to hemin alone. Moderate phenol modification provided an optimal balance between catalytic efficiency and hydrogel integration, whereas excessive modification reduced the performance of the enzyme. Hydrogels containing hemin/BSA-Ph complexes exhibited controllable protein retention and high cytocompatibility (>90%) with mouse fibroblast 10T1/2 cells. These findings demonstrate that hemin/albumin complexes are promising, cost-effective, and cytocompatible alternatives to HRP systems for hydrogel-based biomedical and nonclinical applications.

## 1. Introduction

Hydrogels, three-dimensional networks of water-swollen polymers, have attracted increasing attention in biomedical research because of their biocompatibility and ability to mimic native extracellular environments [1,2,3,4]. Their high water content, tunable mechanical properties, and versatile structures make them suitable for wound healing, drug delivery, and tissue engineering applications. However, conventional hydrogel fabrication often requires chemical crosslinking, which may generate cytotoxic byproducts and hinder the incorporation of sensitive biomolecules [5]. To overcome these limitations, enzymatic crosslinking has emerged as a more biocompatible alternative [6]. Horseradish peroxidase (HRP) has been extensively studied among the various enzymes employed for hydrogel formation [7,8]. HRP facilitates hydrogel formation from aqueous solutions of polymers possessing phenol groups by crosslinking phenol groups in the presence of hydrogen peroxide (H_2_O_2_). This crosslinking occurs under mild physiological conditions, thereby preserving the integrity of cells and biomolecules [7,9,10].

Despite these advantages, HRP-based crosslinking systems have several inherent limitations that restrict their widespread use. HRP is relatively expensive, which increases the overall cost of hydrogel production. Moreover, as a foreign protein, it risks triggering immune responses in clinical applications [11]. Furthermore, its catalytic activity is sensitive to H_2_O_2_ concentrations, as excessive levels of H_2_O_2_ cause enzyme deactivation and reduced control over the gelation process [12,13]. These drawbacks highlight the need to explore alternative catalytic systems that are more stable, cost-effective, cytocompatible, and biocompatible for biomedical applications. From an industrial perspective, horseradish peroxidase remains expensive due to its extraction and purification costs, whereas hemin, a low-molecular-weight iron porphyrin, can be synthesized or derived from abundant hemoglobin sources at a much lower cost [14,15]. This economic advantage provides additional justification for investigating hemin-based catalytic systems as practical and scalable alternatives to HRP.

Hemin, an iron-containing porphyrin structurally analogous to the active site of HRP, has emerged as a promising alternative catalyst for phenol crosslinking during hydrogel formation [16,17,18]. Hemin offers several advantages over HRP, including greater cost-effectiveness and stability in various solvents and under alkaline conditions. However, one major limitation of hemin is its poor solubility, which leads to aggregation in neutral aqueous solutions [19]. Additionally, hemin itself exhibits dose-dependent cytotoxicity [20,21]. Wang et al. reported that the viabilities of mouse neuroblastoma cells after 24 h of culture with 3 and 5 μM hemin were 50% and 20%, respectively [20]. Similarly, Bhoite-Solomon et al. reported that the viabilities of rat ventricular myocytes after 3 h of culture with 5 and 20 μM hemin were 55% and 40%, respectively [21]. These limitations have been addressed by conjugating hemin with other soluble macromolecules [19,22]. For instance, haematin-polymer conjugates such as dendrimer-G3.0-hemin have been shown to effectively catalyze the in situ hydrogelation of phenol-modified polymers under neutral conditions, offering enhanced cytocompatibility and reduced sensitivity to high H_2_O_2_ concentrations [14]. Similarly, conjugating haematin with chitosan and other polymers has improved water solubility, enabling hydrogel formation under physiological pH conditions while maintaining excellent adhesive properties for biomedical applications [15,23].

Recent studies have explored multifunctional hydrogel systems that integrate hemin as a catalyst. For example, hemin-functionalized composite hydrogels have demonstrated improved wound-healing and tissue-regeneration properties [24,25]. Another innovative approach involves incorporating hemin into protein-based nanozyme carriers to enhance stability and bioavailability. Hemin forms a 1:1 complex with albumin, where the aromatic amino acids in albumin coordinate with the Fe center of hemin [26,27]. In the case of bovine serum albumin, this complexation primarily occurs through non-covalent stacking interactions (π–π interactions) and metal coordination between the Fe center of hemin and functional groups on the protein surface. This binding increases hemin solubility and reduces its potential toxicity in the plasma and extracellular environments [26,27]. Such protein-assisted solubilization provides an effective means to overcome free hemin’s inherent aggregation and dissolution limitations, thereby expanding its applicability in biomedical systems.

**Figure 1:**
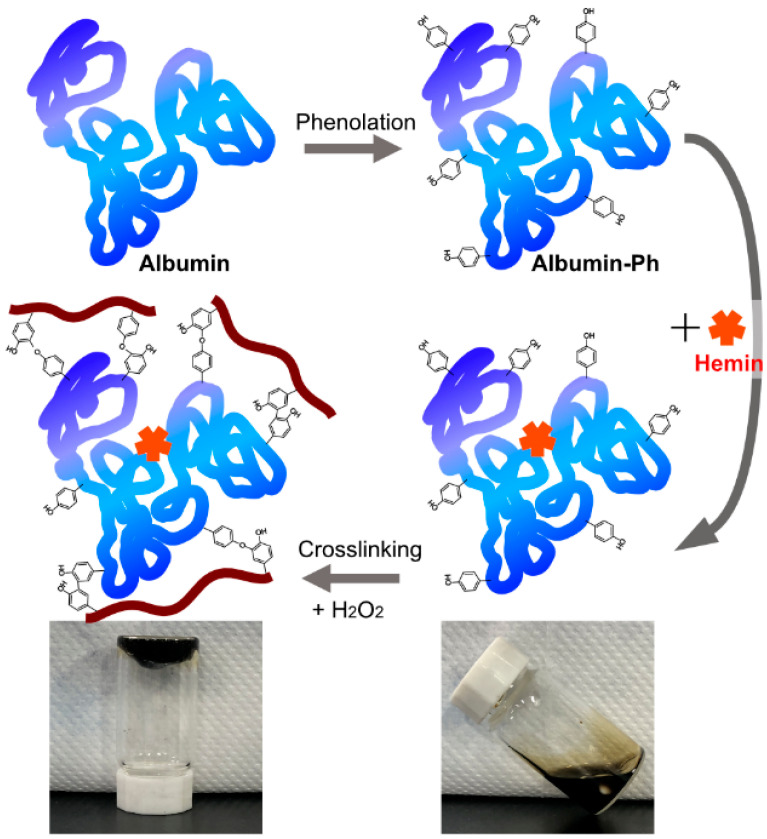
Schematic illustration of phenolated bovine albumin (BSA-Ph) synthesis, hemin/BSA-Ph complex formation, and crosslinking of phenol groups catalyzed by hemin/BSA-Ph in the presence of hydrogen peroxide (H_2_O_2_). Photographs show the precursor solution (**right**) and resulting hydrogel after crosslinking (**left**).

Based on these findings, we aimed to develop a novel hydrogel system in which phenolated albumin not only stabilizes hemin but also serves as a dual-function crosslinker and hydrogel backbone (Figure 1). Albumin is naturally present in the bloodstream and exhibits excellent biocompatibility, making it an ideal biomaterial for biomedical applications [28,29]. Albumin-based hydrogels have gained attention for biomedical applications, including tissue engineering and regenerative medicine [30,31]. Hydrogels consisting of albumin-Ph have been prepared through an HRP-mediated process [32,33]. To the best of our knowledge, there are no reports on hydrogels obtained through hemin-mediated crosslinking.

## 2. Results and Discussion

In this study, we systematically evaluated the effects of complex formation with albumin-Ph on hemin functionality and hydrogelation properties, including gelation time, albumin release kinetics, and cytocompatibility. Phenolated bovine serum albumin (BSA-Ph) with different Ph contents, 8.3 × 10^−5^, 2.9 × 10^−4^, and 4.7 × 10^−4^ mol-Ph/g, denoted as BSA-LPh, -MPh, and -HPh, respectively, were employed.

### 2.1. Formation of Hemin/BSA Complexes

**Figure 2:**
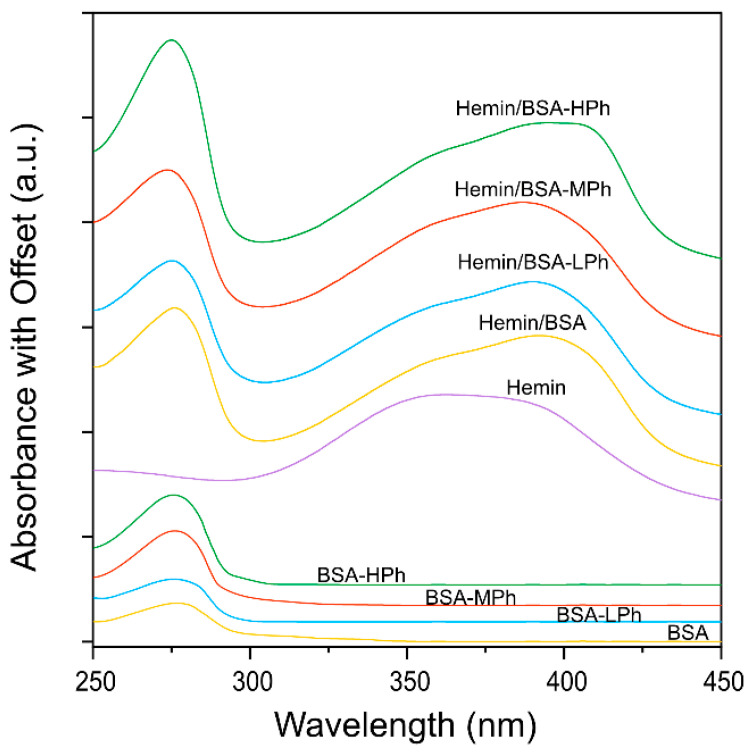
UV–Vis absorbance spectra of BSA, its phenol-modified derivatives (BSA-LPh, -MPh, -HPh), and hemin-containing complexes (hemin, hemin/BSA, hemin/BSA-LPh, hemin/BSA-MPh, hemin/BSA-HPh). All BSA-based samples were measured at the same BSA concentration, whereas hemin-containing samples were measured at the same hemin concentration. Spectra are vertically offset for clarity.

UV–Vis absorbance spectra were recorded for aqueous solutions containing BSA, BSA-Phs, and their mixtures with hemin in the wavelength range of 250–450 nm (Figure 2). All BSA-based samples exhibited a single peak near 280 nm with no absorbance around 400 nm, indicating no spectral overlap with the hemin Soret band, a strong absorption feature of porphyrin-containing compounds [26]. By contrast, hemin alone exhibited a broad Soret band centered near 375 nm. Upon mixing with native BSA, the Soret band became slightly sharper and red-shifted, as previously reported [26]. Interestingly, the hemin/BSA-LPh and -MPh mixtures exhibited a modest blue shift (a shift toward shorter wavelengths), whereas BSA-HPh showed a slight red shift (a shift toward longer wavelengths), indicating different interaction strengths between hemin and BSA-Phs.

These findings suggest that the hemin microenvironment strongly depends on the degree of phenol modification, which alters the π–π stacking and hydrophobic interactions. The modest blue shift observed for BSA-LPh and BSA-MPh suggests weaker interactions or lower encapsulation efficiency, potentially due to lower Ph-mediated binding affinity. Blue shifts in hemin absorption have been reported when the porphyrin ring remains partially exposed to the aqueous phase, reflecting weaker binding and reduced encapsulation within the protein matrices [34]. By contrast, the red shift observed with BSA-HPh implies stronger π–π stacking or hydrophobic interactions between the porphyrin ring of hemin and the densely phenolated albumin surface, stabilizing the hemin in a more conjugated electronic state. These spectral changes collectively support the successful formation of hemin/BSA-Ph complexes with tunable interaction strengths depending on the Ph content.

A slight molar excess of albumin (1.5 mM) over hemin (1.2 mM) was used to prevent hemin aggregation and ensure complete complex formation. Excess free hemin tends to self-associate under neutral aqueous conditions, which decreases its catalytic activity. The additional albumin stabilizes hemin through hydrophobic and π–π interactions, maintaining its solubility and reproducible catalytic behavior.

Further verification of these non-covalent interactions could be achieved using complementary techniques such as fluorescence quenching or isothermal titration calorimetry (ITC), enabling quantitative evaluation of binding constants and energetics.

### 2.2. Catalytic Activity of Hemin/BSA Complexes

To comprehensively evaluate the catalytic performance of the hemin/BSA and hemin/BSA-Ph complexes, two complementary methods were employed: (1) quantification of dityrosine formation from tyramine as a model reaction and (2) measurement of gelation time in phenol-functionalized hydrogel precursors.

**Figure 3:**
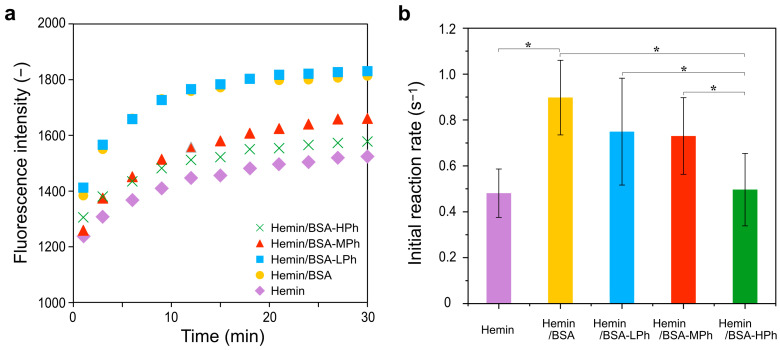
(**a**) Time-dependent fluorescence intensity of dityrosine formed from tyramine in the presence of hemin, hemin/BSA, and phenol-modified hemin/BSA complexes (HPh, MPh, LPh), indicating the catalytic efficiency of each system. (**b**) Comparison of the initial reaction rates calculated from the linear portion of the dityrosine formation curves (until 240 s). Data are presented as mean ± SD *(n* = 10–12). * *p* < 0.05, one-way ANOVA with Tukey’s post hoc test.

As shown in Figure 3a, the time-dependent increase in fluorescence intensity attributed to dityrosine reflected the progression of phenol crosslinking in tyramine by hemin. The hemin/BSA and hemin/BSA-LPh complexes exhibited higher fluorescence intensities over time than hemin alone, indicating enhanced catalytic activity. The initial reaction rates determined from the fluorescence increase during the first 240 s of measurement (Figure 3b) revealed that BSA complexation significantly improved the catalytic efficiency compared to hemin alone (*p* < 0.05). Among the hemin/BSA-Ph complexes, hemin/BSA-LPh preserved activity comparable to that of unmodified hemin/BSA, whereas hemin/BSA-HPh exhibited progressively reduced activity.

**Figure 4:**
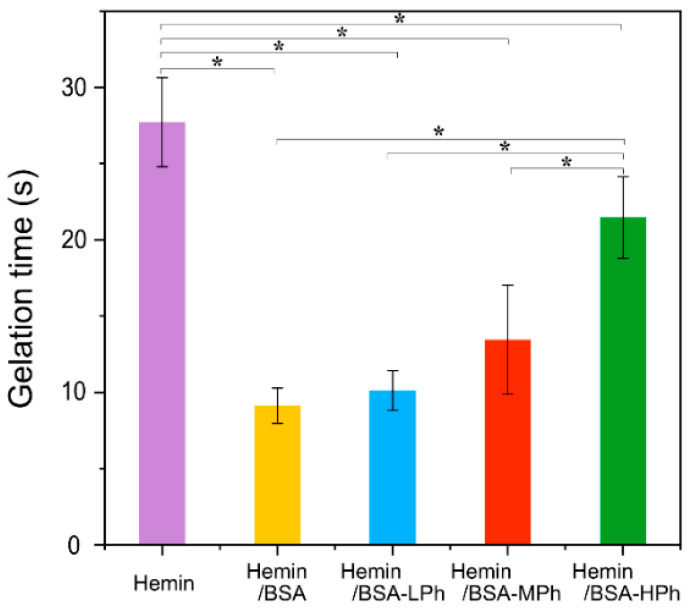
Gelation time for the transition from solution to hydrogel composed of 1 w/v% HA-Ph and 1 w/v% Gelatin-Ph in the presence of 1.2 mM hemin or hemin-BSA complexes (BSA, BSA-LPh, -MPh, and BSA-HPh) with 10 mM H_2_O_2_. The complexation with BSA significantly accelerated gelation, particularly with unmodified BSA and BSA-LPh complexes. Data are presented as mean ± SD (*n* = 3–4). * *p* < 0.05, one-way ANOVA with Tukey’s post hoc test.

Catalytic activity was further assessed by the gelation kinetics of precursor solutions composed of 1 w/v% HA-Ph and 1 w/v% gelatin-Ph in the presence of 1.2 mM hemin complex and 10 mM H_2_O_2_ (Figure 4). While hemin/BSA and hemin/BSA-LPh accelerated gelation, hemin/BSA-MPh and hemin/BSA-HPh delayed gelation, likely because of steric hindrance and reduced substrate accessibility (~14 s and ~22 s, respectively). These results agree with the trends observed in the dityrosine assay using tyramine as the substrate (Figure 3).

Overall, the catalytic activity of hemin was enhanced by albumin complexation but modulated by the degree of phenol substitution, underscoring the importance of tuning chemical modification to balance catalytic accessibility and hydrogel stability. The concordance between the molecular (tyramine dimerization) and macroscopic (hydrogelation) systems demonstrates that the hemin/BSA-Ph complexes function as effective crosslinking catalysts across different phenol-containing substrates. This underscores their potential versatility in both molecular conjugation and hydrogel fabrication.

The enhanced catalytic performance of hemin on complexation with BSA is consistent with previous reports [35]. This enhancement is commonly attributed to non-covalent interactions, such as π–π stacking and coordination between the iron center of hemin and specific amino acid residues in albumin. The catalytic behavior of the hemin/BSA-Ph system can be directly compared to HRP-catalyzed polymer–Ph hydrogels previously reported by Sakai and co-workers [12,13], which exhibited similar gelation times (approximately 10–20 s) under comparable polymer and H_2_O_2_ concentrations. These findings suggest that hemin/albumin complexes achieve catalytic efficiency comparable to that of HRP while providing improved cost-effectiveness and stability. Commercial HRP (≥250 U/mg) typically costs around 1000–1500 USD/g, whereas hemin is available at less than 20 USD/g, supporting the substantial cost advantage of hemin/albumin-based systems for scalable hydrogel fabrication (see [14,15,22]). This economic benefit, combined with the comparable catalytic efficiency demonstrated in this study, highlights the practical utility of hemin as a replacement for HRP in phenol-mediated crosslinking. However, the effects of phenol modification on these interactions have not been thoroughly investigated. Our findings demonstrate that the catalytic behavior of hemin is highly dependent on the degree of phenol substitution in albumin. While moderate phenol modification (BSA-LPh) maintained a favorable balance between catalytic accessibility and gel integration, higher phenol content (BSA-MPh and BSA-HPh) likely imposed steric hindrance or electronic constraints, limiting substrate access and reducing turnover efficiency. Moreover, excessive interactions between hemin and phenol-rich albumin may hinder dynamic substrate exchange, thereby attenuating catalysis.

Taken together, these results suggest that a moderate degree of phenol modification provides an optimal balance between covalent incorporation and catalytic activity of the enzyme. By contrast, over-modification compromises the efficiency, emphasizing the importance of tuning the phenol content in protein-based hydrogel catalysts.

### 2.3. Release of Albumin from Hydrogels

Following the catalytic performance evaluation, we examined how the phenolation of albumin influences its retention within the hydrogels, as albumin retention is critical for hydrogel functionality in biomedical applications such as drug delivery and wound healing. Hydrogels were fabricated from 2% w/v HA-Ph and 1.2 mM hemin/BSA or hemin/BSA-Ph complexes, and albumin release was quantified on days 2, 4, and 9 after incubation.

**Figure 5:**
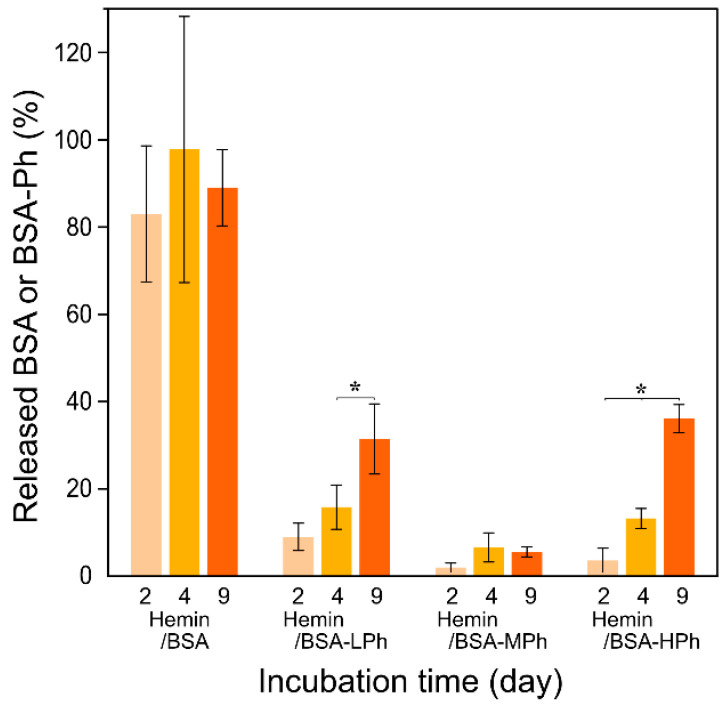
Cumulative release of BSA and phenol-modified BSA (BSA-LPh, -MPh, and-HPh) from hydrogels containing hemin complexes. The percentage of protein released into phosphate-buffered saline (PBS) was measured on days 2, 4, and 9 post implantation. Data are presented as mean ± standard deviation (*n* = 3). * *p* < 0.05, one-way ANOVA with Tukey’s post hoc test.

As shown in Figure 5, the hemin/BSA-containing hydrogels exhibited rapid release, with ~80% of the BSA diffusing from the hydrogels within two days. By contrast, those containing hemin/BSA-Phs showed markedly slower release, with cumulative release levels remaining below 40% even after 9 d. Among them, the hemin/BSA-MPh hydrogel exhibited the lowest release (~5% for 4 d, with little further increase by day 9), suggesting efficient covalent immobilization of BSA-Ph.

Interestingly, as shown in Figure 3 and Figure 4, the hemin/BSA-LPh system, despite having the lowest phenol content, exhibited the fastest catalytic rate and the highest protein release of the phenolated BSA systems. Therefore, the number of phenol groups in BSA-LPh is insufficient to permit efficient crosslinking, resulting in incomplete incorporation. This trade-off highlights the need to optimize the modification levels for specific biomedical applications.

This interpretation aligns with previous studies reporting that moderate biomolecule functionalization enhances hydrogel integration, whereas excessive non-site-selective modifications impair reactivity due to steric effects [36,37]. For example, Li et al. demonstrated the reduced incorporation of over-modified protein–polymer conjugates due to steric hindrance near reactive sites [36]. Such tunability offers a versatile design strategy for tailoring hydrogel performance. BSA-MPh-based hydrogels may be suitable for applications requiring sustained protein retention, such as cell-laden scaffolds and wound dressings. By contrast, unmodified BSA may benefit systems in which early burst release promotes cell spreading and migration. It should be noted that the release of albumin was not intentionally designed or controlled in this study; the observed behavior represents the intrinsic diffusion and retention properties of the hydrogel under the given conditions.

To further verify the structural integrity of the hydrogels, compression tests were performed to evaluate their mechanical properties (Supplementary Figure S1). The Young’s modulus decreased with increasing phenol content, suggesting that higher degrees of phenol modification reduced the crosslinking density and stiffness of the hydrogels. Because the exact phenol contents could not be fully matched among the three preparations, these data are presented as qualitative evidence supporting the inverse correlation between the phenol modification level and the gel stiffness. This trend is consistent with the catalytic and gelation results (Figure 3 and Figure 4), indicating that excessive phenol modification hinders effective crosslinking and leads to the formation of softer hydrogel networks.

### 2.4. Cytocompatibility of Hydrogels

The cytocompatibility of hydrogels containing hemin or hemin/BSA complexes was evaluated using mouse fibroblasts (10T1/2). Cell-laden hydrogels were fabricated from HA-Ph and gelatin-Ph precursor solutions containing 1.2 mM hemin or hemin/BSA(-Ph) complexes, followed by culturing for up to 7 d.

**Figure 6:**
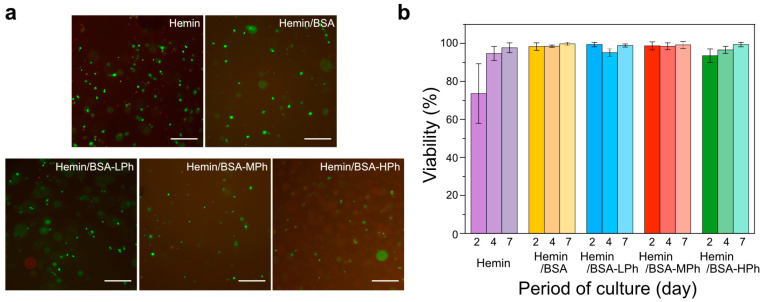
Cell viability in hydrogels containing hemin or hemin/BSA complexes. (**a**) Representative fluorescence images of encapsulated cells in the hydrogels on day 2 stained with calcein-AM (live, green) and propidium iodide (dead, red). Scale bars: 300 µm. (**b**) Cell viability over 7 d of culture, showing consistently high viability across all hydrogel formulations. Data are presented as the mean ± SD (*n* = 3–4).

As shown in Figure 6a, live/dead staining on day 2 revealed mostly viable 10T1/2 cells in hydrogels containing hemin/BSA or hemin/BSA-Ph complexes, whereas hydrogels with hemin alone displayed more dead cells. Quantitative analysis (Figure 6b) consistently showed reduced viability (73.6%) for hemin alone, whereas all albumin-containing systems maintained viability of more than 90%. This initial decrease in cell viability in the hydrogel prepared using hemin alone is consistent with the well-documented cytotoxicity of hemin, which generates reactive oxygen species and induces membrane damage [38,39]. The subsequent recovery of viability in the hemin group (days 4 and 7) is likely due to the binding of hemin to the albumin present in fetal bovine serum (FBS), which sequesters hemin and reduces its oxidative toxicity [40,41].

By contrast, when hemin was pre-complexed with albumin or BSA-Phs, no early drop in viability was observed, indicating that albumin binding stabilized hemin and prevented its cytotoxic effects from the outset [40,41]. Among the phenol-modified variants (BSA-LPh, -MPh, and-HPh), no significant differences in cell viability were detected, confirming that the degree of phenol modification did not adversely affect cytocompatibility. This contrasts with the catalytic performance and protein retention (Figure 3, Figure 4 and Figure 5), which were strongly influenced by the phenol content.

Fibroblasts were selected as a representative adherent cell model for the initial cytocompatibility assessment because they are widely used to evaluate the biocompatibility of hydrogel scaffolds and are recommended by international standards (ISO 10993-5:2020) for in vitro cytotoxicity testing [42]. Although only one cell type was examined in this study, future investigations will include other relevant cell types, such as endothelial and stem cells, to further validate the biocompatibility of the hemin/BSA-Ph hydrogel system.

These results demonstrate that the complexation of hemin with albumin effectively suppresses hemin-induced cytotoxicity by stabilizing the hemin structure and reducing reactive oxygen species generation. Importantly, phenol modification of BSA enables control over catalytic efficiency and protein retention without compromising cell viability, underscoring the suitability of hemin/BSA-Ph complexes for hydrogel-based biomedical applications, such as cell-laden scaffolds and wound-healing materials.

### 2.5. Limitations and Perspective

This study establishes the feasibility of hemin/BSA-Ph complexes as catalysts for hydrogel formation and provides a foundation for future translational applications. While this work primarily focused on BSA as a model protein, its success demonstrates the potential of the hemin/protein-mediated crosslinking strategy. Although BSA provides a convenient and well-characterized platform, it differs from human serum albumin in terms of sequence and structure, which may influence the ligand-binding behavior and stability [43,44]. For clinical translation, it is essential to validate the system using HSA. To address this point, we have provided supplemental preliminary data showing that phenolated human serum albumin also forms hydrogels via hemin-mediated crosslinking (Supplemental Figure S2).

BSA-Ph-based hydrogels also hold substantial potential for non-clinical applications. Importantly, HRP-catalyzed polymer-Ph hydrogel systems have been reported for applications in tissue engineering in vitro [6,7], providing proof-of-concept that enzymatically crosslinked phenol-containing hydrogels are useful as cell-laden scaffolds, 3D culture matrices, and drug screening platforms. In line with these precedents, BSA-Ph/hemin-based hydrogels are expected to serve as versatile in vitro tools for regenerative medicine research and pharmacological assays, although their direct clinical application requires further development.

Future studies will expand the characterization of these hydrogels, including degradation kinetics of the gels and cell-type-dependent response, to strengthen their translational potential. Furthermore, although the catalytic efficiency of hemin was shown to be tunable by adjusting the degree of phenol modification, the underlying molecular mechanisms, such as the balance of π–π stacking, steric hindrance, and electron transfer efficiency, remain to be elucidated in greater detail, potentially through molecular dynamics simulations or spectroscopic analyses [41,45].

The hemin/BSA-Ph platform is an attractive alternative to enzyme-based hydrogel systems. Compared to HRP, hemin provides greater chemical stability and is less expensive, and its performance can be modulated by protein complexation and chemical modification. This tunability creates opportunities for biomedical and broader applications in soft materials science, such as bio-inspired adhesives and catalytic scaffolds. The design space for hemin/protein-mediated hydrogels can be expanded by systematically integrating experimental and computational approaches, paving the way for in vitro research tools and long-term translation strategies.

In addition, FTIR spectroscopy could further confirm phenol–phenol crosslinking by detecting the decrease in O–H stretching and the appearance of characteristic C–O–C and aromatic C–C bands, providing molecular-level evidence of covalent bond formation within the hemin/BSA-Ph hydrogel network [46].

Collectively, this work demonstrates the dual catalytic and structural role of albumin in hemin-mediated hydrogel formation and establishes a versatile platform for future biomedical, biofabrication, and materials applications.

## 3. Conclusions

This study demonstrated that a hemin-albumin complex and its phenol-modified derivatives constitute a versatile catalytic system for hydrogel formation, offering a tunable alternative to HRP. Hydrogels incorporating hemin/BSA-Ph complexes exhibited controllable albumin release, with BSA-MPh showing prolonged retention of albumin. All albumin-containing formulations (hemin/BSA and hemin/BSA-Ph complexes) maintained high cytocompatibility (>90%) with the mouse fibroblast 10T1/2 cells. Overall, the hemin/BSA-Ph complexes are promising, cost-effective, and biocompatible alternatives to HRP for phenol-mediated hydrogel formation, with potential biomedical and non-clinical applications. In addition to biomedical applications, such as wound healing and tissue regeneration, these systems are suitable as in vitro research tools, including 3D culture scaffolds and drug testing platforms. This work broadens the utility of hemin-based catalytic systems and provides a framework for the rational design of protein–phenol conjugate hydrogels with tunable catalytic and biological performances.

## 4. Materials and Methods

### 4.1. Materials

Hemin (porcine), BSA, *N*-hydroxysuccinimide (NHS), anhydrous tetrahydrofuran (THF), anhydrous dichloromethane (DCM), and dimethylformamide (DMF) were obtained from Wako Pure Chemical Industries (Osaka, Japan). Sodium hyaluronic acid (HA, Mw ~ 800 kDa), gelatin Type B, 3-(4-hydroxyphenyl)propionic acid (HPPA), tyramine hydrochloride, and 1-ethyl-3-(3-dimethylaminopropyl)carbodiimide hydrochloride (WSCD) were obtained from Kiwpie (Tokyo, Japan), Sigma-Aldrich (St. Louis, MO, USA), Tokyo Chemical Industry (Tokyo, Japan), Chem-Impex International (Wood Dale, IL, USA), and Peptide Institute (Osaka, Japan), respectively.

### 4.2. Synthesis of Phenolated Polymers

#### 4.2.1. Synthesis of 3-(4-Hydroxyphenyl)propionic Acid N-Hydroxysuccinimide Ester

HPPA (1.66 g), NHS (1.84 g), and WSCD (2.50 g) were dissolved in THF/DCM (1:1, 180 mL) under a nitrogen atmosphere and stirred for 18 h at room temperature (22 ± 2 °C). The mixture was successively washed with water, saturated sodium bicarbonate, and saturated sodium chloride solutions. The organic layer was dried over anhydrous sodium sulfate, filtered, and concentrated using silica gel (8.1 g). Crude HPPA-NHS was purified by silica gel column chromatography (Isolera One, Biotage, Uppsala, Sweden) and dried under vacuum.

#### 4.2.2. Synthesis of BSA-Ph

BSA was dissolved in 50 mM phosphate-buffered saline (PBS; pH 7.4) at 1.7 mg/mL. HPPA-NHS was separately dissolved in DMF at concentrations of 2, 10, or 20 mM. The BSA and HPPA-NHS solutions were mixed at a volume ratio of 8:1 and stirred overnight at room temperature (22 ± 2 °C). The reaction mixture was dialyzed (MWCO 12–14 kDa) against distilled water for 48 h with frequent water changes and subsequently lyophilized. The resulting phenolated BSA samples were designated as BSA-LPh, -MPh, and -HPh, corresponding to increasing HPPA-NHS concentrations. The phenol content in BSA-Ph was quantified by UV–Vis spectrophotometry. The absorbance of a 0.1 w/w% aqueous solution was measured at 275 nm, and the phenol concentration was calculated from a calibration curve constructed with tyramine as the standard compound. The phenol contents in BSA-LPh, -MPh, and -HPh were 8.3 × 10^−5^, 2.9 × 10^−4^, and 4.7 × 10^−4^ mol-Ph/g, respectively.

#### 4.2.3. Synthesis of HA-Ph

Phenolated HA (HA-Ph) was synthesized as previously described [47]. HA-Na (1.0 g) was dissolved in 100 mL of 0.1 M MES buffer (pH 6.0). Tyramine hydrochloride (0.348 g), NHS (0.115 g), and WSCD (0.1916 g) were sequentially added. The reaction mixture was stirred overnight at room temperature (22 ± 2 °C). After adjusting the pH to 8.6 with 1 M NaOH, the reaction mixture was precipitated in acetone, washed with 80% ethanol, stirred overnight in 100% ethanol, and vacuum-dried. The phenol content in the resultant HA-Ph was 6.9 × 10^−5^ mol-Ph/g.

#### 4.2.4. Synthesis of Gelatin-Ph

Phenolated gelatin (Gelatin-Ph) was synthesized according to a previously reported method [12]. Gelatin (40 g) was dissolved in a mixture of 600 mL distilled water and 400 mL DMF. HPPA (13.3 g), NHS (12.8 g), and WSCD (15.2 g) were added, and the mixture was stirred overnight at room temperature (22 ± 2 °C). The product was purified by dialysis against water for 3 d and then lyophilized. The phenol content in the resultant gelatin-Ph was 4.5 × 10^−4^ mol-Ph/g.

### 4.3. Preparation of Hemin/Albumin-Ph Complex

Hemin (0.4 g) was dissolved in 50 mL of a 0.274 M NaOH solution. The pH of the solution was adjusted to 8.7 by the sequential addition of 1 mL of 1 M HCl and 1 mL of 0.2 M HCl. Subsequently, 33 mL of triple-concentrated phosphate-buffered saline (PBS) was added, and the solution was further diluted with distilled water to a final volume of 100 mL, resulting in a hemin concentration of 6.0 mM in PBS. The hemin solution was mixed with PBS containing 1.5 mM albumin-Ph to prepare a solution containing 1.2 mM each of hemin and albumin-Ph. The mixture was stirred with a magnetic stirrer at room temperature (22 ± 2 °C) for 3 h and then filtered through a 0.2 μm pore size filter. The UV-visible absorption spectra of the filtered solutions were measured using a UV-vis spectrophotometer (UV-2600, Shimadzu, Kyoto, Japan). The formation of the hemin/albumin-Ph complex was confirmed by a shift in the Soret band maximum from approximately 385 nm for hemin alone to approximately 392 nm upon binding to albumin [26]. BSA was used as a control for BSA-Ph.

### 4.4. Catalytic Activity of Hemin/Albumin-Ph Complex

The catalytic activity of the hemin/BSA-Ph complex was determined by quantifying the increase in fluorescence associated with dityrosine formation from tyramine (excitation: 315 nm, emission: 410 nm), which reflects the crosslinking of phenolic (Ph) moieties. Mixtures of tyramine and hemin, hemin/BSA, or hemin/BSA-Ph complexes were dispensed into the wells of a 96-well plate. An aqueous H_2_O_2_ solution was then added to each well to achieve final concentrations of 8 μM hemin, hemin/albumin, or hemin/albumin-Ph complex, 27 mM tyramine, and 13 mM H_2_O_2_. The fluorescence intensity derived from the dityrosine was monitored as described above.

Catalytic activity was evaluated by determining the hydrogelation time of a mixture of HA-Ph and gelatin-Ph solutions. Mixtures of HA-Ph and gelatin-Ph containing hemin, hemin/albumin, or hemin/albumin-Ph complexes were dispensed into the wells of a 48-well microplate. An aqueous H_2_O_2_ solution was then added to each well and stirred using a magnetic stir bar to achieve final concentrations of 1.0 w/v% for both HA-Ph and gelatin-Ph, 1.2 mM hemin, hemin/albumin, hemin/albumin-Ph complex, and 10 mM H_2_O_2_. The concentration of H_2_O_2_ (10 mM) was empirically optimized in preliminary tests, which confirmed that this concentration allowed uniform gel formation without visible precipitation. The total volume of the solution in each well was 0.3 mL per well. The hydrogelation time was defined as the time at which the magnetic stirrer bar stopped rotating, as confirmed by the formation of a solid-like network.

### 4.5. Release of BSA and BSA-Phs from Hydrogels

PBS containing 2 w/v% HA-Ph and 1.2 mM hemin/BSA or the hemin/BSA-Ph complex (0.05 mL) was filled into plastic vessels 8 mm in diameter and 2 mm in height. The vessels were then placed in air containing 16 ppm of H_2_O_2_ for 1 h to obtain disk-shaped hydrogels with a height of 1 mm. The resulting hydrogels were immersed in 4 mL PBS, and an aliquot of the solution was collected on days 2, 4, and 9. The BSA and BSA-Ph contents in the aliquots were quantified using the Bradford Protein Assay (Bio-Rad, Hercules, CA, USA), according to the manufacturer’s instructions.

### 4.6. Encapsulation of Cells in Hydrogels

Mouse fibroblast 10T1/2 cells, obtained from the Riken Cell Bank (Ibaraki, Japan), were encapsulated in hydrogels composed of 1 w/v% HA-Ph, 1 w/v% gelatin-Ph, and 1.2 mM hemin, hemin/BSA, or hemin/BSA-Ph complexes. Precursor solutions were prepared to contain 2.5 × 10^5^ cells/mL and dispensed (50 μL) onto polydimethylsiloxane substrates. Gelation was induced by exposing the gel to an atmosphere containing 16 ppm H_2_O_2_ for 20 min. The resulting cell-laden hydrogels were cultured in Dulbecco’s modified Eagle’s medium (DMEM) containing 10% (v/v) FBS with 1 mg/mL catalase (Fujifilm-Wako, Osaka, Japan). After 30 min and 1 h, the medium was replaced with fresh catalase-containing DMEM and incubated overnight. The following day, the culture medium was replaced with catalase-free DMEM for subsequent experiments. Cell viability was evaluated by live/dead staining with calcein-AM (live, green) and propidium iodide (PI; dead, red). Fluorescent images were obtained using a fluorescence microscope (BZ-9000, Keyence, Osaka, Japan), and the percentage of viable cells was calculated as the ratio of calcein-positive cells to the total number of cells.

### 4.7. Statistical Analysis

All experiments were performed at least in triplicate and the results are reported as the mean ± SD. Statistical significance was determined using a one-way ANOVA with Tukey’s post hoc test. A *p*-value < 0.05 was considered significant.

## Data Availability

The original contributions presented in the study are included in the article/Supplementary Materials. Further inquiries can be directed to the corresponding authors.

## Supplementary Materials

The following supporting information can be downloaded at: https://www.mdpi.com/article/10.3390/gels11110912/s1. Figure S1: Mechanical properties of hydrogels prepared by exposing solutions containing 1 w/v% HA-Ph and 1 w/v% gelatin-Ph with 1.2 mM hemin or hemin/BSA complexes (BSA, BSA-1.4Ph, BSA-1.9Ph, and BSA-3.1Ph) to air containing 16 ppm H_2_O_2_ for 20 min. Data are presented as mean ± SD (*n* = 3). *p* < 0.05, one-way ANOVA with Tukey’s post hoc test. BSA-1.4Ph, BSA-1.9Ph, and BSA-3.1Ph correspond to BSA-Ph samples with phenolic group contents of 1.4, 1.9, and 3.1 × 10^−4^ mol-Ph/g, respectively; Figure S2: Photographs of a hemin/HSA-Ph solution (Left) before and (Right) after adding H_2_O_2_.

## Abbreviations

The following abbreviations are used in this manuscript:

HRP	horseradish peroxidase
BSA	bovine serum albumin
BSA-Ph	phenolated bovine serum albumin
H_2_O_2_	hydrogen peroxide
PBS	phosphate-buffered saline
HA	hyaluronic acid
HA-Ph	phenolated hyaluronic acid
Gelatin-Ph	phenolated gelatin
