# Supplementary material for: Dual-Function Role of Phenolated Albumin in Hemin-Mediated Hydrogel Formation
Source: Gels. 2025 Nov 15;11(11):912. doi: 10.3390/gels11110912 (PMC12652777; doi:10.3390/gels11110912)
Supplement: Supplementary file 1 [file gels-11-00912-s001.zip › gels-3942221-supplementary.pdf]

Supporting information

Article

Dual-Function Role of Phenolated Albumin in Hemin-Mediated Hydrogel Formation

Shinji Sakai [1,*], Yuki Kitatani [1], Maasa Shiba [2], Thotage Asanka Vishwanath [1], Kelum Chamara Manoj Lakmal Elvitigala [1], Wildan Mubarok [1] and Kousuke Moriyama [2,*]

1 Department of Materials Engineering Science, Graduate School of Engineering Science, The University of Osaka, Toyonaka 560-8531, Japan; asanka.vish@cheng.es.osaka-u.ac.jp (T.A.V.); kelum@cheng.es.osaka-u.ac.jp (K.C.M.L.E.); wildanmubarok@cheng.es.osaka-u.ac.jp (W.M.)
2 Department of Chemical and Biological Engineering, National Institute of Technology, Sasebo College, 1-1 Okishin-cho, Sasebo 857-1193, Japan
* Correspondence: sakai@cheng.es.osaka-u.ac.jp (S.S.); moriyama@sasebo.ac.jp (K.M.)

Method: Mechanical Properties

The stiffness of the synthesized hydrogels was determined in terms of Young's modulus using a material tester (EZ-SX, Shimadzu, Kyoto, Japan). First, PBS containing 1 w/v% HA-Ph, 1 w/v% gelatin-Ph, and 1.2 mM hemin, hemin/BSA, or hemin/BSA-Ph complexes was filled into a 35 mm dish and exposed to air containing 16 ppm of H_2O_2 for 1 h to obtain disk-shaped hydrogels with an average height of ~1.5 mm. Three BSA-Phs with different phenolic group content, 1.4, 1.9 and 3.1 $\times 10^{-4}$ mol-Ph/g were used. Then, each composite hydrogel was compressed at 6 mm/s using a 3 mm probe. Next, Young's modulus was calculated to determine the stiffness of the prepared hydrogel using the linear compression strain of 1–10% of the stress–strain curve.

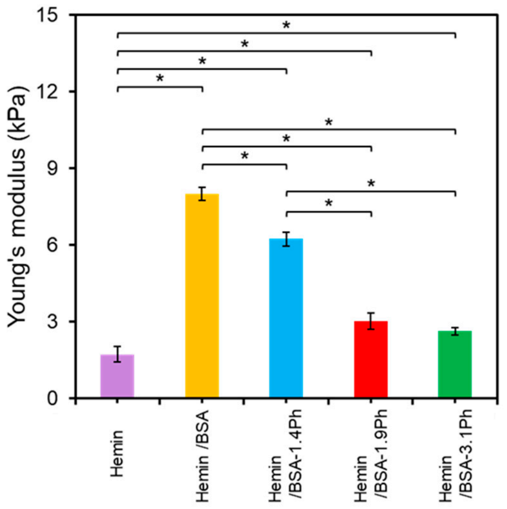

Figure S1. Mechanical properties of hydrogels prepared by exposing solutions containing 1 w/v% HA-Ph and 1 w/v% gelatin-Ph with 1.2 mM hemin or hemin/BSA complexes (BSA, BSA-1.4Ph, BSA-1.9Ph, and BSA-3.1Ph) to air containing 16 ppm H_2O_2 for 20 min. Data are presented as mean ± SD ($n = 3$). $p < 0.05$, one-way ANOVA with Tukey's post hoc test. BSA-1.4Ph, BSA-1.9Ph, and BSA-3.1Ph correspond to BSA-Ph samples with phenolic group contents of 1.4, 1.9, and 3.1 × 10^{-4} mol-Ph/g, respectively.

Method: Synthesis of hemin/HSA-Ph

Human serum albumin (HSA, 2 g) was dissolved in 100 mL of MES buffer (final concentration: 0.3 mM). Hemin (19.8 mg) was separately dissolved in 1 mL of DMSO (final concentration: 30 mM). The HSA and hemin solutions were mixed at a molar ratio 1:1 and stirred for 24 h at room temperature. Subsequently, tyramine (2 g), N-hydroxysuccinimide (NHS, 0.334 g), and 1-ethyl-3-(3-dimethylaminopropyl)carbodiimide (EDC, 0.566 g) were sequentially added to the mixture and stirred for an additional 24 h. The reaction mixture was concentrated by ultrafiltration and dialyzed (MWCO 14 kDa) against distilled water to remove unreacted components. The dialyzed solution was then lyophilized to obtain hemin/HSA-Ph. The phenol content of resulting hemin/HSA-Ph was determined to be 1.72×10^{-4} mol-Ph/g.

Gelation tests were conducted in glass tubes with an inner diameter of 10 mm. Hemin/HSA-Ph was dissolved in PBS (pH 7.4). To 0.18 mL of the hemin/HSA-Ph solution, 0.020 mL of H_2O_2 was added and gently mixed by pipetting. The final concentrations of hemin/HSA-Ph and H_2O_2 were 8 w/w% and 15 mM, respectively.

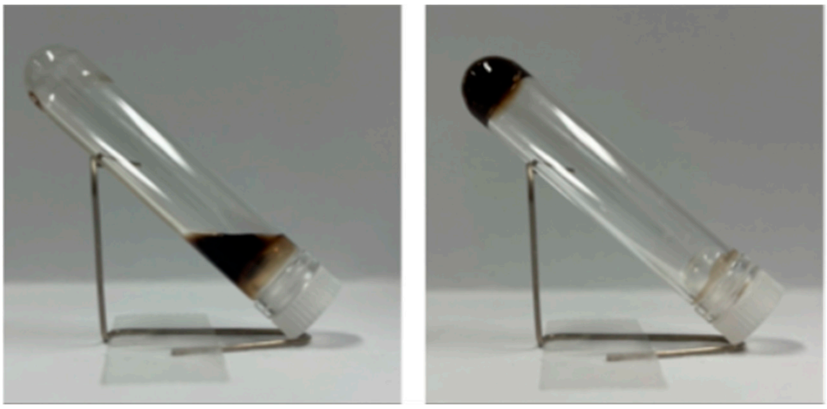

Figure S2. Photographs of a hemin/HSA-Ph solution (Left) before and (Right) after adding H_2O_2.
